# Corrigendum to “Primary bilateral breast lymphoma in an elder male patient”

**DOI:** 10.1155/tbj/9821303

**Published:** 2025-09-19

Y. Bozkaya, F. Oz Puyan, and B. Bimboga, “Primary bilateral breast lymphoma in an elder male patient,” *The Breast Journal* 25, no. 5 (2019): 1008-1009, https://doi.org/10.1111/tbj.13394.

In the article titled “Primary bilateral breast lymphoma in an elder male patient,” there was a spelling error in author Busem Binboga's name in the author list, where Busem Bimboga should have read Busem Binboga. The corrected author list and affiliation list should be as follows:

Yakup Bozkaya^1^, Fulya Oz Puyan^2^, Busem Binboga^2^

^1^Clinic of Medical Oncology, Edirne State Hospital, Edirne, Turkey

^2^Department of Pathology, Faculty of Medicine, Trakya University, Edirne, Turkey

We apologize for this error.

